# Network connectome analysis of multi omics data identifies molecular markers of recurrence and grade progression in meningioma

**DOI:** 10.3389/fonc.2026.1745505

**Published:** 2026-03-02

**Authors:** Jeong-An Gim, Hyun Jun Jo, Woo Keun Kwon, Chang Hwa Ham, Hae Won Roh, Wonki Yoon, Jong Hyun Kim, Taek Hyun Kwon, Joonho Byun

**Affiliations:** 1 Department of Medical Science, Soonchunhyang University, Asan, Chungcheongnam, Republic of Korea; 2 Department of Neurosurgery, Korea University Guro Hospital, Korea University College of Medicine, Seoul, Republic of Korea

**Keywords:** meningioma, network connectome, recurrence, analysis, LINC01397

## Abstract

**Background:**

Meningiomas are usually benign, but some behave aggressively with early recurrence. Histopathological grading alone often fails to predict outcomes. We developed a network connectome and clustering framework that integrates DNA methylation, RNA-seq, and proteomic data to identify molecular interaction patterns linked to recurrence and grade progression.

**Methods:**

Using genome-wide methylation, transcriptomic, and proteomic profiles, we constructed multi-layer connectome networks representing inter-omic correlations. Nodes and edges were analyzed by centrality and clustering metrics to detect key molecular modules associated with clinical outcomes.

**Results:**

Distinct network clusters differentiated recurrent and higher-grade meningiomas from indolent ones. A total of 29 methylation, 32 gene, and 33 protein features were significantly related to recurrence; 70, 61, and 56 features were linked to grade progression. Recurrent tumors showed increased inter-omic connectivity and altered hub distributions. LINC01397 emerged as a recurrent hub across omic layers, suggesting its role as a potential unified biomarker.

**Conclusion:**

Our connectome-based multi-omics analysis reveals that meningioma aggressiveness is driven by coordinated molecular interactions rather than single-omic alterations. This systems-level approach provides a compact, data-driven framework for predicting recurrence and grade, supporting precision risk stratification in clinical practice.

## Introduction

Meningiomas are the most common brain tumors in adults (1). Although many behave indolently, approximately 20% show aggressive features such as rapid growth, brain invasion, and a high likelihood of recurrence. Most of these clinically challenging cases fall within WHO grade 2 or 3, which remain difficult to predict accurately using histology alone (2).

Recent efforts have focused on identifying molecular alterations that improve prognostication. The 2021 WHO classification incorporated genetic features such as CDKN2A/B homozygous deletion and TERT promoter mutation, reflecting the growing recognition that molecular markers can refine grading and clinical decision making (3, 4). Advances in DNA methylation profiling have further improved risk stratification, supporting the development of integrated molecular–morphological classifications for more personalized management (5–9).

However, identifying disease-associated genes through large cohorts or laboratory experiments remains costly and time-consuming. Computational approaches therefore play an important role in prioritizing candidate genes. In this study, we applied a network connectome approach—originally developed to characterize connectivity patterns in neuroscience—to explore molecular interactions across DNA methylation, RNA expression, and protein expression using publicly available datasets from cBioPortal (10).

Previous studies of meningioma recurrence have mainly used dimensionality reduction and clustering to define molecular subgroups (5, 11). While informative, such methods do not capture system-level interactions. To address this limitation, we used network connectome analysis to integrate multi omics features and identify molecular modules associated with recurrence and WHO grade progression. Matched methylation, transcriptomic, and proteomic data from the same patients enabled a comprehensive assessment of differentially altered regions, genes, and proteins within a unified analytic framework.

## Materials and method

### Data acquisition

**Figure 1:**
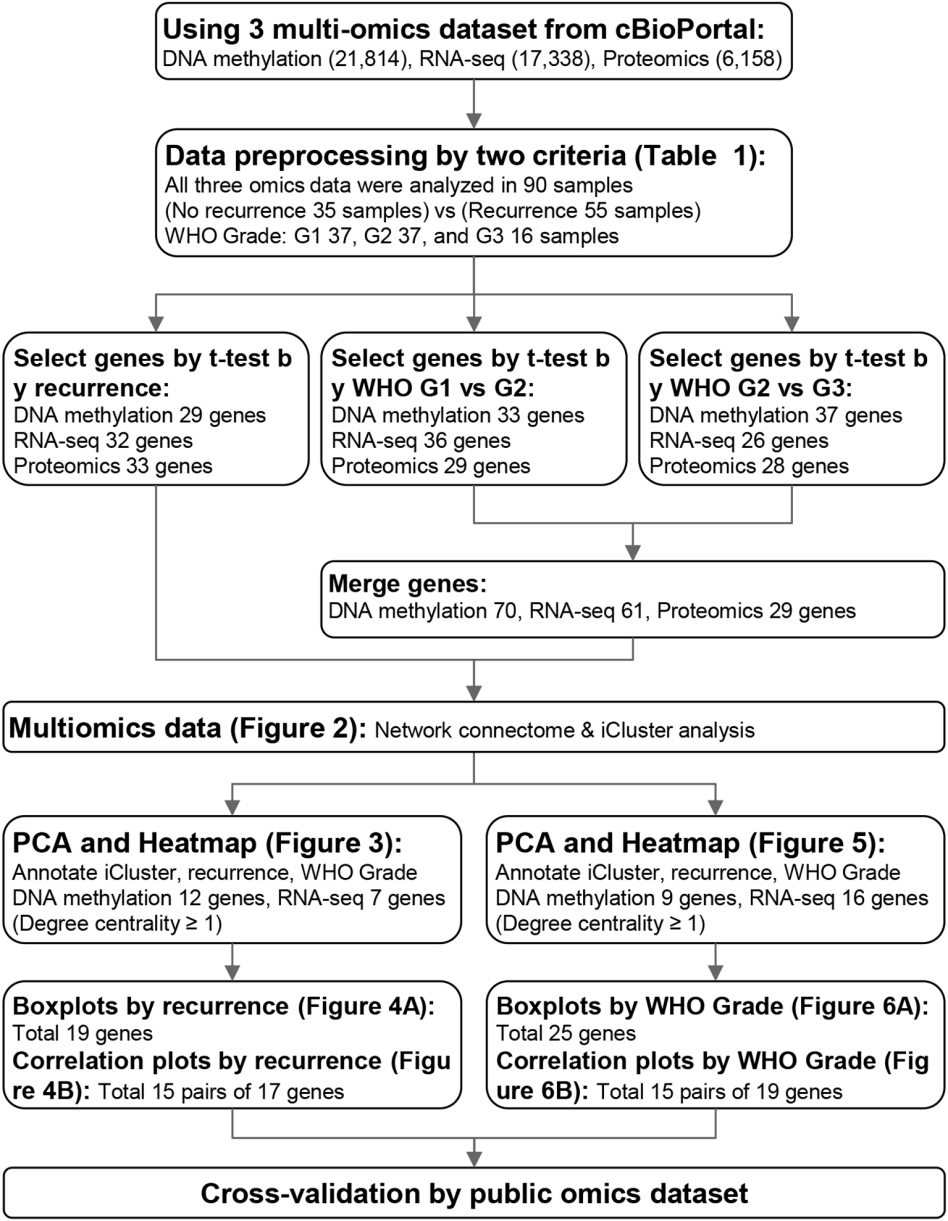
Process of this study.

This study was approved by the Institutional Review Board of Soonchunhyang University (Approval Number: 202405-SB-049) and conducted in accordance with the Declaration of Helsinki. DNA methylation, RNA-seq, proteomic, and clinical datasets of intracranial meningiomas were obtained from cBioPortal. All analyses were performed in R version 4.4.1. The overall workflow is summarized in **Figure 1**.

### Identification of DMRs, DEGs, and DEPs

Normalized DNA methylation (21,814 CpG sites), RNA-seq (17,338 genes), and proteomic (6,158 proteins) data were analyzed. Ninety patients with matched multi-omics profiles were included. Patients were grouped by recurrence status and WHO grade (Grade 1 = 37, Grade 2 = 37, Grade 3 = 16). Differentially methylated regions (DMRs), differentially expressed genes (DEGs), and differentially expressed proteins (DEPs) were identified using two-group *t*-tests, and significant features were visualized with volcano plots and heatmaps. Selection thresholds for each omics layer are summarized in **Table 1**.

**Table 1:** Sample numbers and threshold list (PV; p-value, FC; fold change).

Criteria	Recurrence		G1 vs G2		G2 vs G3	
	No	Yes	G1	G2	G2	G3
Sample no.	35	55	37	37	37	16
Threshold	**PV**	**FC**	**PV**	**FC**	**PV**	**FC**
Methylation	0.001	0.18	0.001	0.2	0.005	0.18
RNA-seq	0.001	1.7	0.001	1.8	0.01	1.6
Proteomics	0.001	1	0.001	1	0.01	1

In the three omics datasets from a single patient, differentially methylated regions (DMRs), differentially expressed genes (DEGs), and differentially expressed proteins (DEPs) were identified based on the following criteria. PV, p-value; FC, fold change.

### Network connectome and clustering analysis

The identified DMRs, DEGs, and DEPs were integrated into a unified multi-omics matrix. Principal component analysis (PCA) and multi-omics clustering were performed to explore overall data structure. For connectome construction, Pearson correlation matrices were constructed, and features with correlation coefficients ≥ 0.7 (p = 1.63 × 10^−14^) for recurrence analysis or ≥ 0.75 (p = 1.79 × 10^−17^) for WHO grade analysis were retained for connectome generation. Node importance was evaluated by degree, betweenness, closeness, and eigenvector centralities, and connectome maps were visualized to highlight molecular interaction hubs. Gene Ontology enrichment was performed to explore biological processes related to significant modules.

### Cross-validation with public datasets

Independent public datasets were retrieved from the NCBI GEO database using the keyword “meningioma.” Datasets containing methylation, transcriptomic, or proteomic profiles were processed using the same analytic pipeline to validate DMRs, DEGs, and DEPs associated with recurrence and grade. Overlapping genes between discovery and validation sets are listed in **Supplementary Table 1**.

## Results

### Feature selection from DMRs, DEGs, and DEPs

From the multi-omics datasets, 21,814 CpG sites, 17,338 genes, and 6,158 proteins were analyzed. Based on the selection criteria summarized in **Table 1**, 29 DMRs, 32 DEGs, and 33 DEPs were identified as significantly associated with recurrence (p < 0.001). These features were visualized using volcano plots and heatmaps (**Supplementary Figure 1**).

For WHO grade progression, differential analyses between Grades 1–2 and 2–3 revealed 70 DMRs, 61 DEGs, and 56 DEPs meeting the same statistical thresholds (**Table 1**; **Supplementary Figure 2**). Distinct hypo- and hypermethylation as well as expression patterns were observed across grades, reflecting progressive molecular changes related to tumor aggressiveness.

### Network connectome analysis

**Figure 2:**
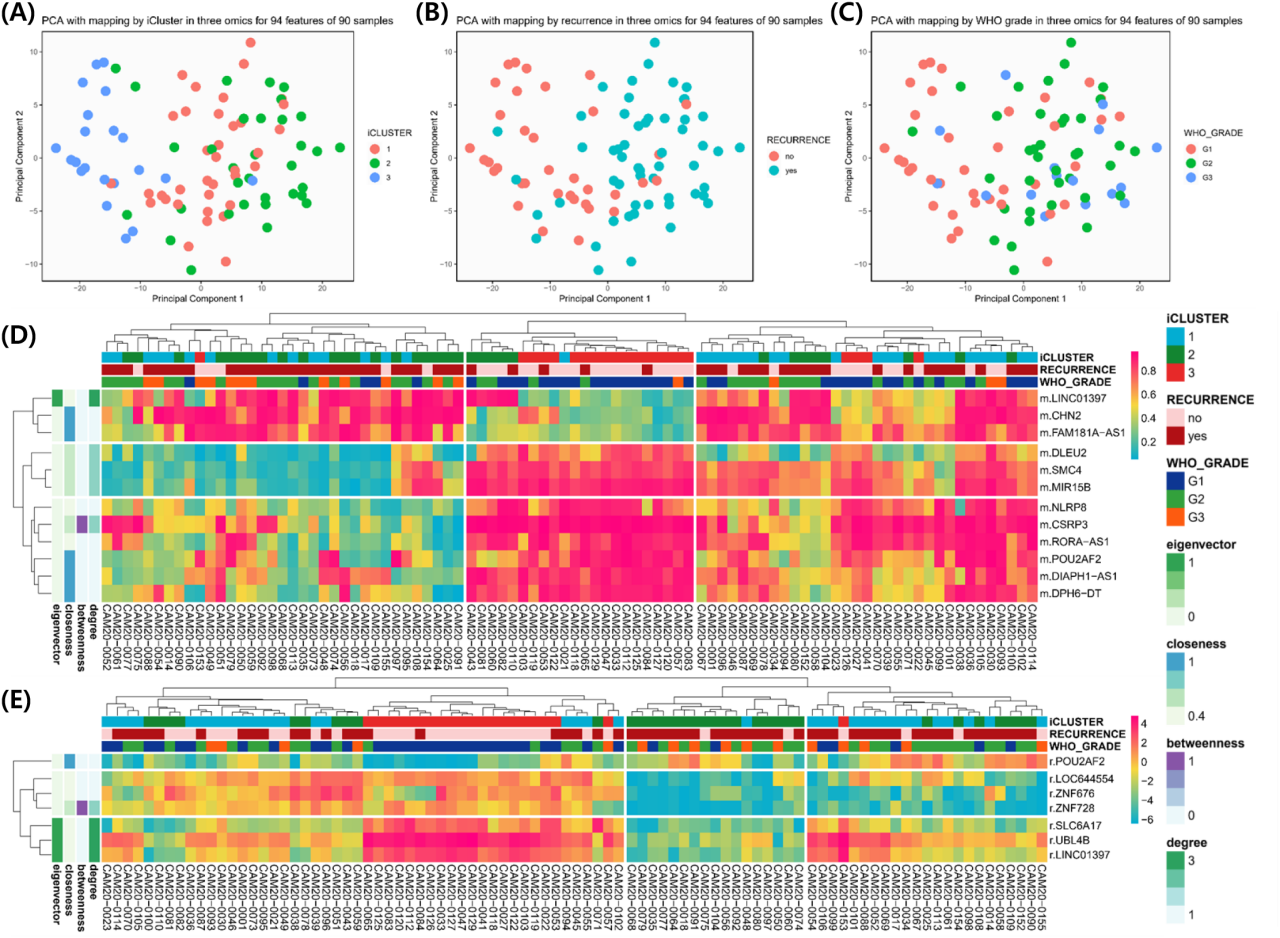
**(A–C)** Principal component analysis (PCA) plots showing sample distribution according to iCluster, recurrence status, and WHO grade. **(D, E)** Heatmaps displaying hierarchical clustering of molecular features across samples with associated annotations.

To characterize molecular interactions underlying these features, we integrated DMRs, DEGs, and DEPs into multi-layer networks. The recurrence network consisted of 94 features (29 DMRs, 32 DEGs, 33 DEPs), and the grade network included 187 features (70 DMRs, 61 DEGs, 56 DEPs). Pearson correlation matrices were constructed, and features with correlation coefficients ≥ 0.7 (recurrence) or ≥ 0.75 (grade) were retained for connectome generation. Graph-based analysis revealed seven distinct molecular clusters for recurrence and six for WHO grade (**Figure 2**). Centrality mapping identified key network hubs, with LINC01397 emerging as a recurrent central node across omics layers. Gene ontology enrichment indicated involvement of extracellular matrix organization, cell adhesion, and signal transduction pathways (**Table 2**).

**Table 2:** Gene ontology (GO) analysis of total genes detected in network connectome analysis.

Class	Term	P-value	Combined score	Genes
Recurrence	striated muscle hypertrophy (GO:0014897)	0.00509	1318.68	CSRP3
Recurrence	cardiac muscle hypertrophy (GO:0003300)	0.006781	890.6522	CSRP3
Recurrence	alanine transport (GO:0032328)	0.007625	760.9634	SLC6A17
Recurrence	proline transport (GO:0015824)	0.007625	760.9634	SLC6A17
Recurrence	glycine transport (GO:0015816)	0.007625	760.9634	SLC6A17
Recurrence	response to muscle stretch (GO:0035994)	0.007625	760.9634	CSRP3
Recurrence	modification-dependent macromolecule catabolic process (GO:0043632)	0.010155	520.8353	UBL4B
Recurrence	mitotic chromosome condensation (GO:0007076)	0.012679	389.3804	SMC4
Grade	histone H3-K4 demethylation (GO:0034720)	0.008718	657.6336	KDM5D
Grade	daunorubicin metabolic process (GO:0044597)	0.009958	547.8577	AKR1C3
Grade	doxorubicin metabolic process (GO:0044598)	0.009958	547.8577	AKR1C3
Grade	aminoglycoside antibiotic metabolic process (GO:0030647)	0.009958	547.8577	AKR1C3
Grade	ketone biosynthetic process (GO:0042181)	0.009958	547.8577	AKR1C3
Grade	protein hexamerization (GO:0034214)	0.011196	467.165	SPAST
Grade	postsynaptic membrane assembly (GO:0097104)	0.011196	467.165	NLGN4Y
Grade	axonal transport of mitochondrion (GO:0019896)	0.011196	467.165	SPAST
Grade	sequestering of actin monomers (GO:0042989)	0.012433	405.5533	TMSB4Y
Grade	quinone metabolic process (GO:1901661)	0.012433	405.5533	AKR1C3
Grade	regulation of actin filament length (GO:0030832)	0.012433	405.5533	TMSB4Y
Grade	presynaptic membrane assembly (GO:0097105)	0.012433	405.5533	NLGN4Y
Grade	primary alcohol catabolic process (GO:0034310)	0.012433	405.5533	AKR1C3
Grade	presynaptic membrane organization (GO:0097090)	0.013668	357.1009	NLGN4Y
Grade	positive regulation of endothelial cell apoptotic process (GO:2000353)	0.013668	357.1009	AKR1C3
Grade	cyclooxygenase pathway (GO:0019371)	0.013668	357.1009	AKR1C3
Grade	response to corticosteroid (GO:0031960)	0.013668	357.1009	AKR1C3
Grade	cellular response to prostaglandin stimulus (GO:0071379)	0.013668	357.1009	AKR1C3
Grade	postsynapse assembly (GO:0099068)	0.013668	357.1009	NLGN4Y
Grade	progesterone metabolic process (GO:0042448)	0.013668	357.1009	AKR1C3
Grade	retinal metabolic process (GO:0042574)	0.013668	357.1009	AKR1C3

### Clustering and multi-omics integration

**Figure 3:**
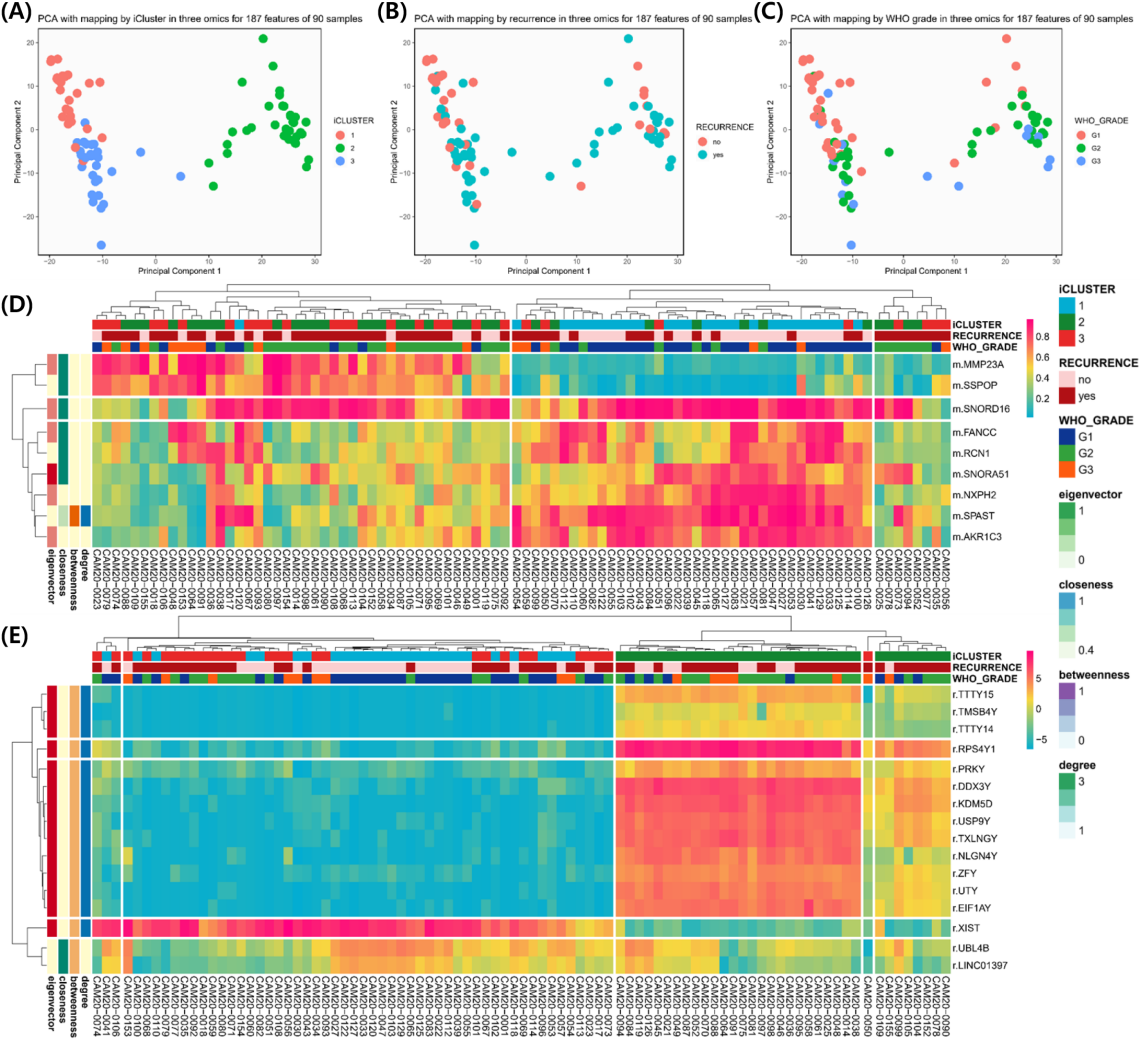
Principal component analysis (PCA) results for 90 samples and heatmap for 19 features used in network connectome analysis. As a result of PCA, annotation according to iCluster **(A)**, recurrence **(B)**, and WHO Grade **(C)** for each 90 plots. A total of 19 features were used in the network connectome analysis according to recurrence, and two heatmaps were presented for each of 12 CpG sites **(D)** and seven genes **(E)**.

**Figure 4:**
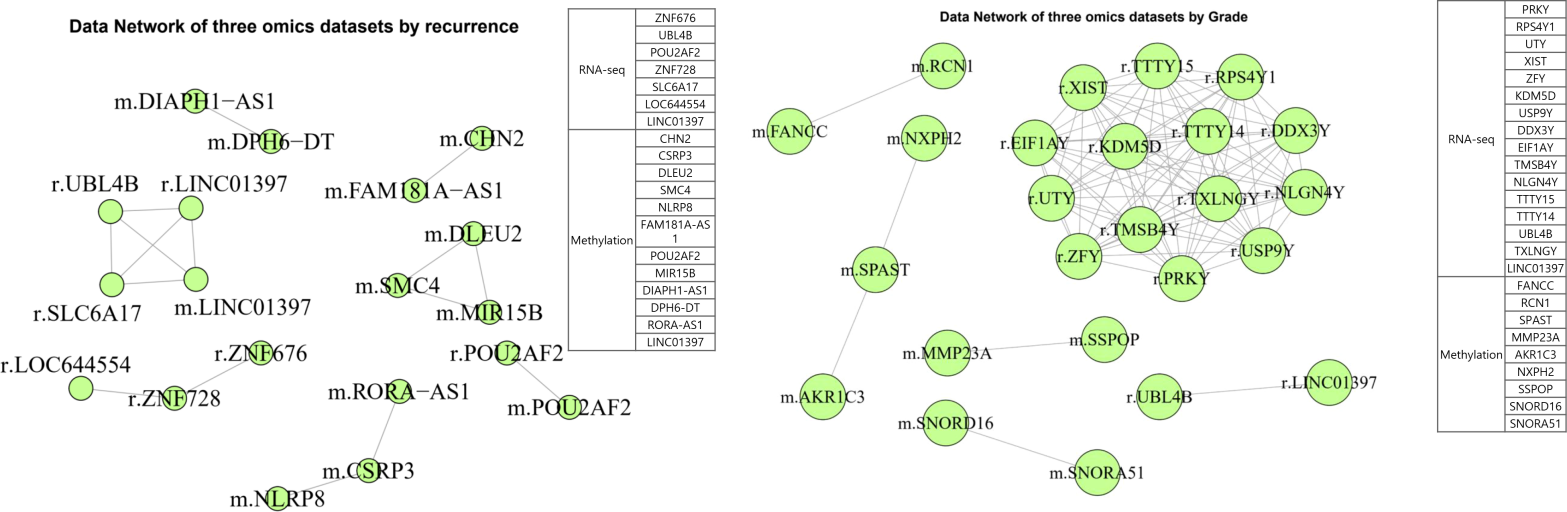
Among 90 patient samples, statistical significance is presented for 37, 37 and 16 Grade 1, 2 and 3 patients, and all satisfied p-value < 0.05. In the network connectome, 15 correlation plots between two features with the same gene symbol and genes belonging to both DEG and DMR and nodes connected to each other was presented. Red points and lines mean no recurrence, and jade points and lines mean recurrence group.

**Figure 5:**
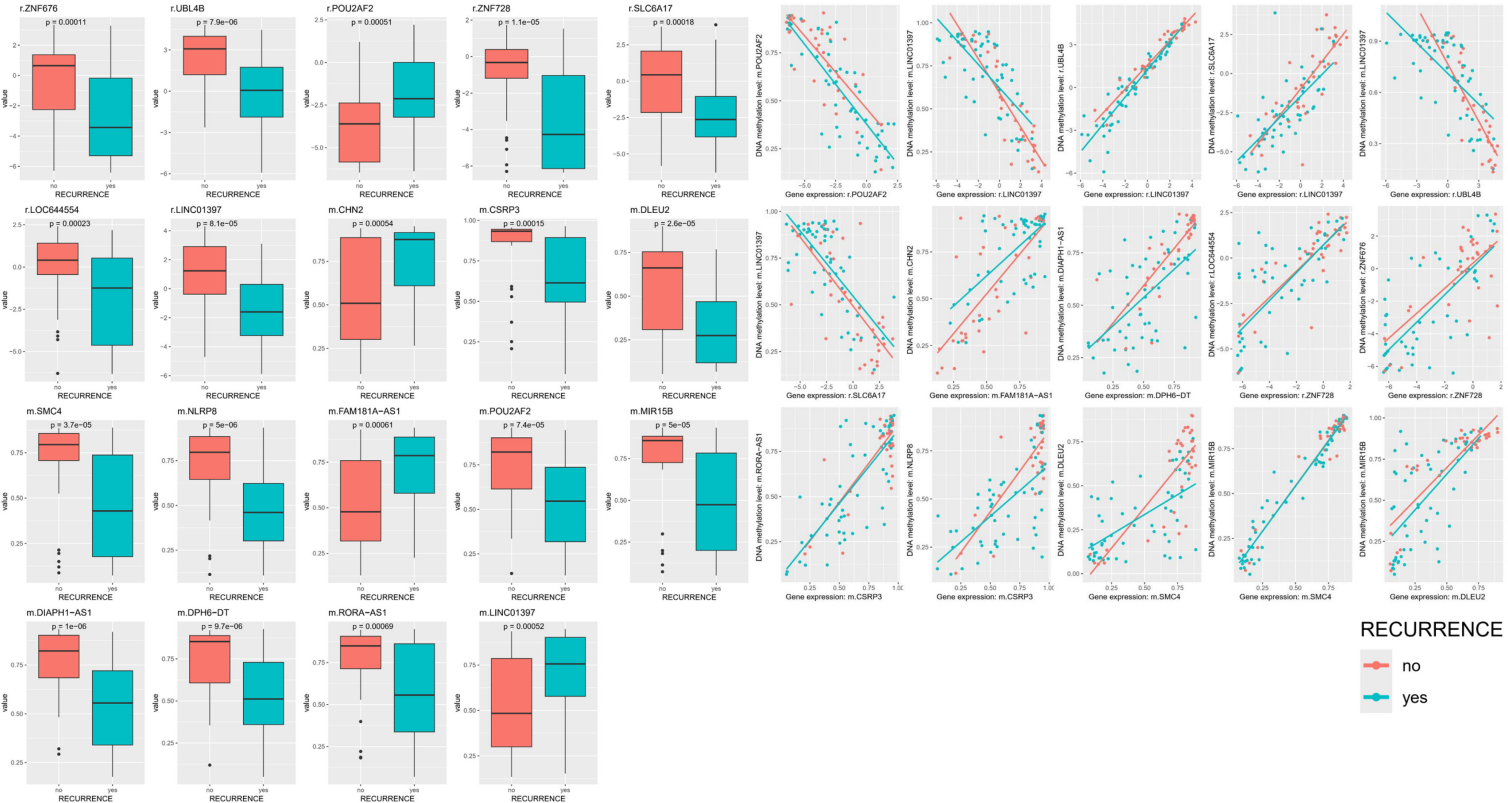
Boxplot according to recurrence for 25 features analyzed in the network connectome. Among 90 patient samples, statistical significance is presented for 37, 37 and 16 Grade 1, 2 and 3 patients, and all satisfied p-value < 0.05. In the network connectome, 15 correlation plots between two features with the same gene symbol and genes belonging to both DEG and DMR and nodes connected to each other was presented. Red points and lines mean no recurrence, and jade points and lines mean recurrence group.

**Figure 6:**
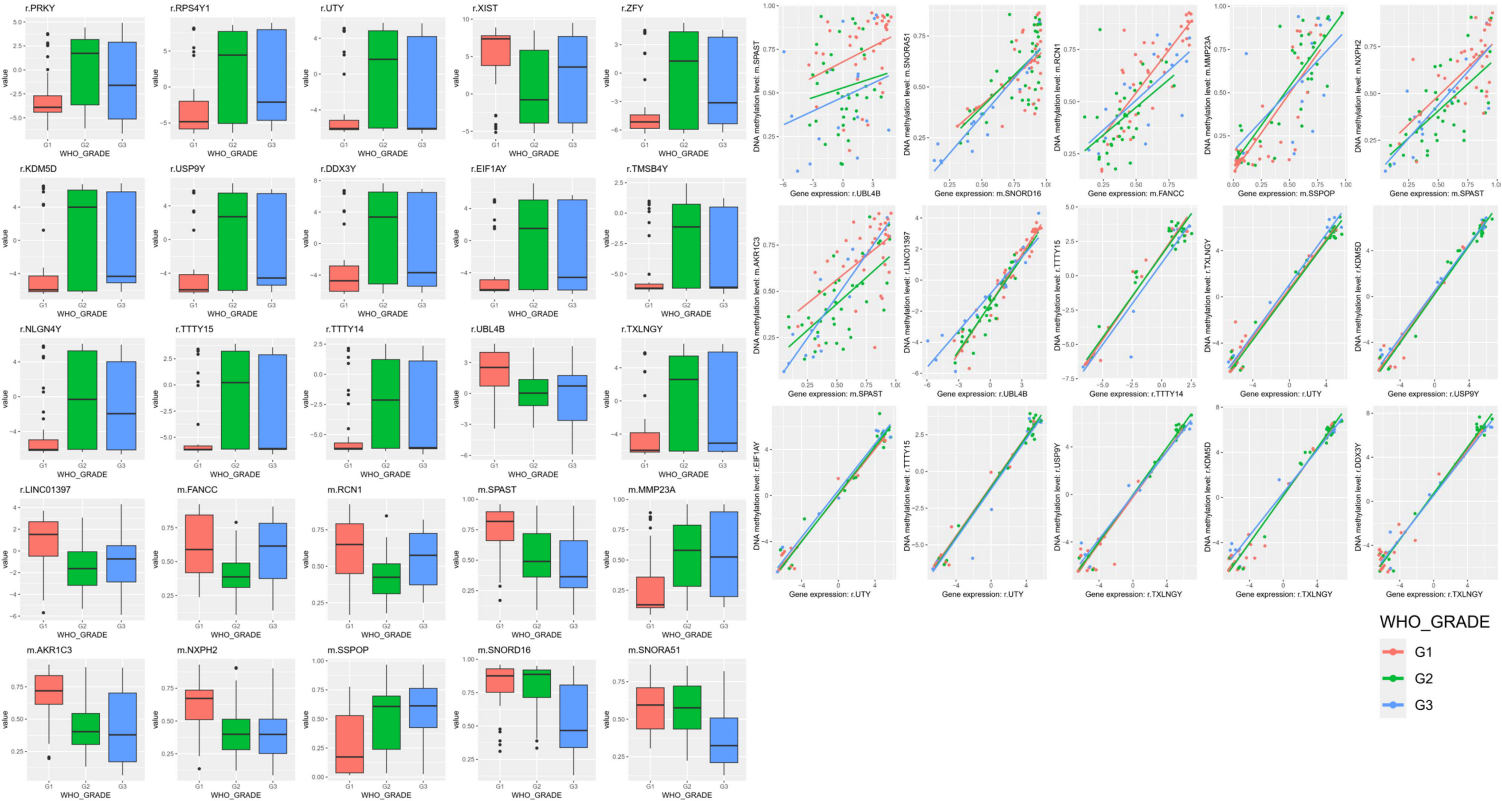
Among 90 patient samples, statistical significance is presented for 37, 37 and 16 Grade 1, 2 and 3 patients, and all satisfied p-value < 0.05. In the network connectome, 15 correlation plots between two features with the same gene symbol and genes belonging to both DEG and DMR and nodes connected to each other was presented. Red points and lines mean no recurrence, and jade points and lines mean recurrence group.

PCA and iCluster analyses were performed using integrated methylation, transcriptomic, and proteomic data from 90 patients. Three molecular clusters were identified (**Figure 3A**). Recurrence status showed clear separation along the first principal component (PC1; **Figure 3B**), while WHO grade separation was less distinct (**Figure 3C**). Cluster 3 predominantly represented nonrecurrent tumors and was characterized by hypomethylation of LINC01397, CHN2, and FAM181A-AS1 with corresponding upregulation of LINC01397 and UBL4B (**Figures 3D, E**). Boxplots demonstrated hypermethylation and reduced expression of LINC01397 in recurrent tumors (**Figure 4**). In contrast, cluster 2 was enriched for recurrent and Grade 2 tumors, showing hypomethylation of MMP23A and SSPOP. Progressive methylation and expression shifts of SPAST, NXPH2, SNORD16, and SNORA51 were associated with increasing WHO grade (**Figures 5**, **6**).

### Cross-validation with public omics datasets

Thirteen public omics datasets were analyzed for validation, including five DNA methylation, seven transcriptomic, and one proteomic dataset. DMRs, DEGs, and DEPs associated with recurrence and grade were reanalyzed using the same thresholds, and overlapping genes with our discovery set were summarized (**Supplementary Table 1**). Consistent differential patterns were observed for several key genes, including CHN2 (methylation), SYNPO2, GSTM5, TCEAL2, MRAP2, SOX11 (gene expression), and KCNMA1, ALPL, SYNPO2, LEPR, GSTM5 (protein expression). Boxplots demonstrated concordant expression or methylation trends across datasets (**Supplementary Figure 5**), supporting the reproducibility of our findings.

## Discussion

Genomic and epigenomic studies have repeatedly shown that meningiomas with the same WHO grade can still behave very differently in the clinic. Well known markers such as SMARCE1, BAP1, KLF4/TRAF7, TERTpromoter mutations, and loss of CDKN2A/B with reduced H3K27me3 expression illustrate how molecular alterations can refine prognostic assessment beyond traditional histology (3, 4, 12). With this in mind, our study set out to explore whether publicly available multi omics data could be reexamined through a different analytical lens, using a network connectome approach to identify signals related to recurrence and grade progression.

The intention was not simply to list differentially expressed or methylated genes, but to understand how these alterations might relate to one another across methylation, transcription, and protein expression. Although connectome analysis has traditionally been used in neuroscience to map connectivity between brain regions, we adapted the concept to examine correlations among molecular features at the genomic and proteomic level. By doing so, we were able to highlight clusters of coordinated changes that would have been difficult to appreciate with single omics analyses alone.

Among the features uncovered, LINC01397, CHN2, and FAM181A-AS1 consistently appeared in central positions within the connectome. LINC01397 showed clear hypermethylation together with reduced expression in recurrent tumors, a pattern that raises the possibility of a tumor suppressive function. CHN2 and FAM181A-AS1 demonstrated similarly structured epigenetic and transcriptional changes, and although these genes have been discussed in other diseases, their relevance to meningioma has not been described before (13–16).

Additional genes such as KCNMA1 and LEPR, which have known links to more aggressive tumor biology, showed concordant trends in external datasets, adding confidence to their relevance (17, 18).

Taken together, these findings suggest that the molecular changes associated with recurrence and grade advancement are not isolated events. Instead, they may represent interconnected shifts involving chromatin regulation, signaling pathways, and cytoskeletal organization, echoing mechanisms proposed in earlier studies. In this sense, the connectome provides a starting point for future mechanistic research that examines not only individual genes but also the relationships among them.

### Implications for future research and clinical practice

The patterns observed here point to several directions for future work. First, larger and independently collected multi omics cohorts will be needed to confirm whether the signatures identified in this study are robust and reproducible. Genes such as LINC01397, CHN2, and FAM181A-AS1 are particularly strong candidates for further investigation, given their consistent associations with recurrence and grade.

From a clinical perspective, the integration of genomic and epigenomic profiling into routine assessment may eventually help refine diagnosis and guide individualized management. Methylation based markers are especially appealing because of their biological stability and the practicality of incorporating them into clinical workflows. Nevertheless, translating these findings into clinical practice will require validation in more diverse populations and prospective studies that assess their predictive value in real time.

Overall, the present study demonstrates that examining multi omics data through a network connectome framework can reveal patterns that remain hidden when each dataset is analyzed independently. This approach offers a broader view of tumor biology and may ultimately contribute to more precise risk stratification and improved outcomes for patients with meningioma.

This study has several limitations. Survival analysis and formal predictive performance metrics were not performed, reflecting the exploratory design and reliance on publicly available multi-omics datasets with limited longitudinal clinical information. In addition, recurrence and WHO grade were used as surrogate clinical endpoints, which may not fully capture patient outcomes. Validation in larger, well-annotated cohorts and prospective studies will be necessary to confirm the biological and clinical relevance of the identified molecular features.

## Conclusion

This study shows that meningioma recurrence and grade progression arise from coordinated molecular shifts across methylation, transcription, and protein expression. Using a network connectome approach, we identified integrative molecular patterns and several promising biomarker candidates. These findings point toward the value of multi omics analyses in refining prognosis and may support future efforts to develop more precise, personalized strategies for managing meningioma.

## Data Availability

The datasets presented in this study can be found in online repositories. The names of the repository/repositories and accession number(s) can be found in the article/**Supplementary Material**.

## Supplementary material

Supplementary Figure 1 Visualization of differentially methylated regions (DMRs) by recurrence as a volcano plot (A) and heatmap (B). From selected 29 DMRs, nine regions showed a hypermethylated pattern in the recurrence group. Visualization of differentially expression genes (DEGs) by recurrence as a volcano plot (C) and heatmap (D). A total of 32 DEGs are composed of seven highly expressed genes and 25 less expressed genes in the recurrence group. Visualization of differentially expression proteins (DEPs) by recurrence as a volcano plot (E) and heatmap (F). The number of DEPs were 33, and 10 proteins were more detected in the recurrence group.

Supplementary Figure 2 Heatmap of all input features for network analysis. Total 94 features consist of 29 differentially methylated regions (DMRs), 32 differentially expression genes (DEGs), 33 differentially expression proteins (DEPs).

Supplementary Figure 3 Heatmap of all input features for network analysis. Total 187 features consist of 70 differentially methylated regions (DMRs), 61 differentially expression genes (DEGs), 56 differentially expression proteins (DEPs). Each DMR, DEG, and DEP was the union of two comparisons, Grade 1 vs 2, and 2 vs 3.

Supplementary Figure 4 Visualization of differentially methylated regions (DMRs) by Grade 1 vs 2 and 2 vs 3. From the comparison of Grade 1 vs 2, total 33 DMRs were selected. In Grade 2, 10 regions were hypermethylated and visualized as a volcano plot (A). Total 37 DMRs were selected by comparing Grade 2 vs 3, and 27 regions were hypermethylated in Grade 3 (B). The union of DMRs obtained from the two comparisons was 70, which was visualized as a heatmap (C). Visualization of differentially expression genes (DEGs) by Grade 1 vs 2 and 2 vs 3. In two comparisons, 36 and 26 DEGs were selected, and 19 and six genes were higher expressed in higher grade (D and E). The union of DEGs were 61, and each expression level was provided as heatmap (F). Differentially expression proteins (DEPs) were selected by grade, Grade 1 vs 2 and 2 vs 3. Total 29 and 28 DEPs were selected, and 22 and nine proteins were more detected in higher grade (G and H). The union of DEPs were 56, which was visualized as a heatmap (I).

Supplementary Figure 5 Visualization of DMRs, DEGs, and DEPs cross-validated genes from public omics data. Expression or methylation patterns for genes found more than six times in the total dataset (ALPL found more than two times for proteins) were visualized as boxplots (ns means not significance, *p < 0.05, **p < 0.01, ***p < 0.001, ****p < 0.0001).
