# Supplementary material for: Network connectome analysis of multi omics data identifies molecular markers of recurrence and grade progression in meningioma
Source: Front Oncol. 2026 Mar 2;16:1745505. doi: 10.3389/fonc.2026.1745505 (PMC12989379; doi:10.3389/fonc.2026.1745505)
Supplement: Supplementary Figure 1 — Visualization of differentially methylated regions (DMRs) by recurrence as a volcano plot (A) and heatmap (B). From selected 29 DMRs, nine regions showed a hypermethylated pattern in the recurrence group. Visualization of differentially expression genes (DEGs) by recurrence as a volcano plot (C) and heatmap (D). A total of 32 DEGs are composed of seven highly expressed genes and 25 less expressed genes in the recurrence group. Visualization of differentially expression proteins (DEPs) by recurrence as a volcano plot (E) and heatmap (F). The number of DEPs were 33, and 10 proteins were more detected in the recurrence group. [file DataSheet1.pdf]

(E)

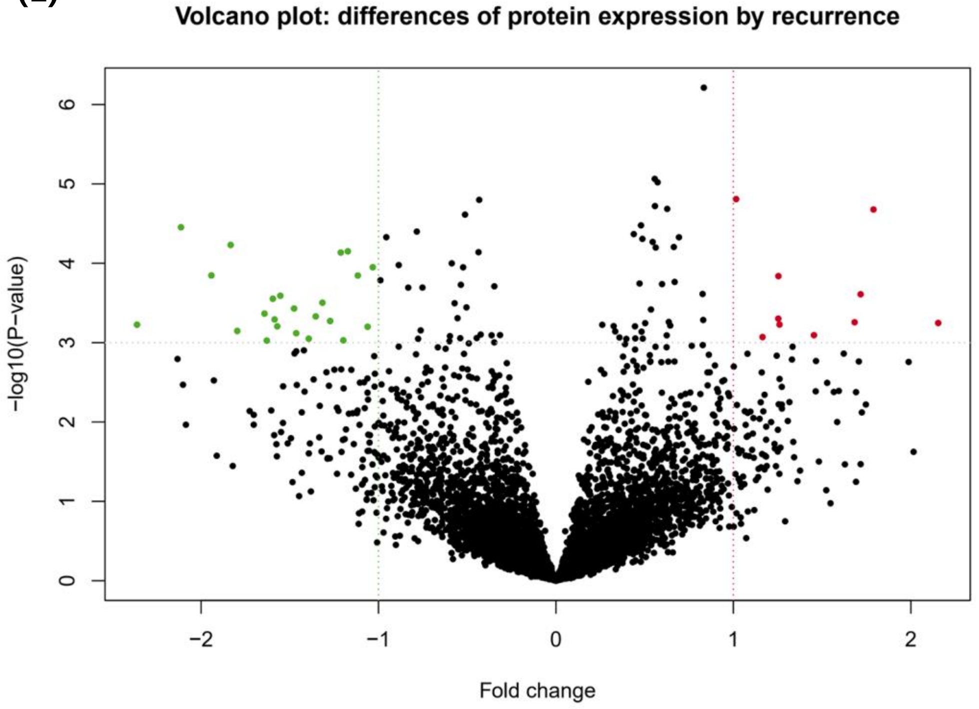

(F)

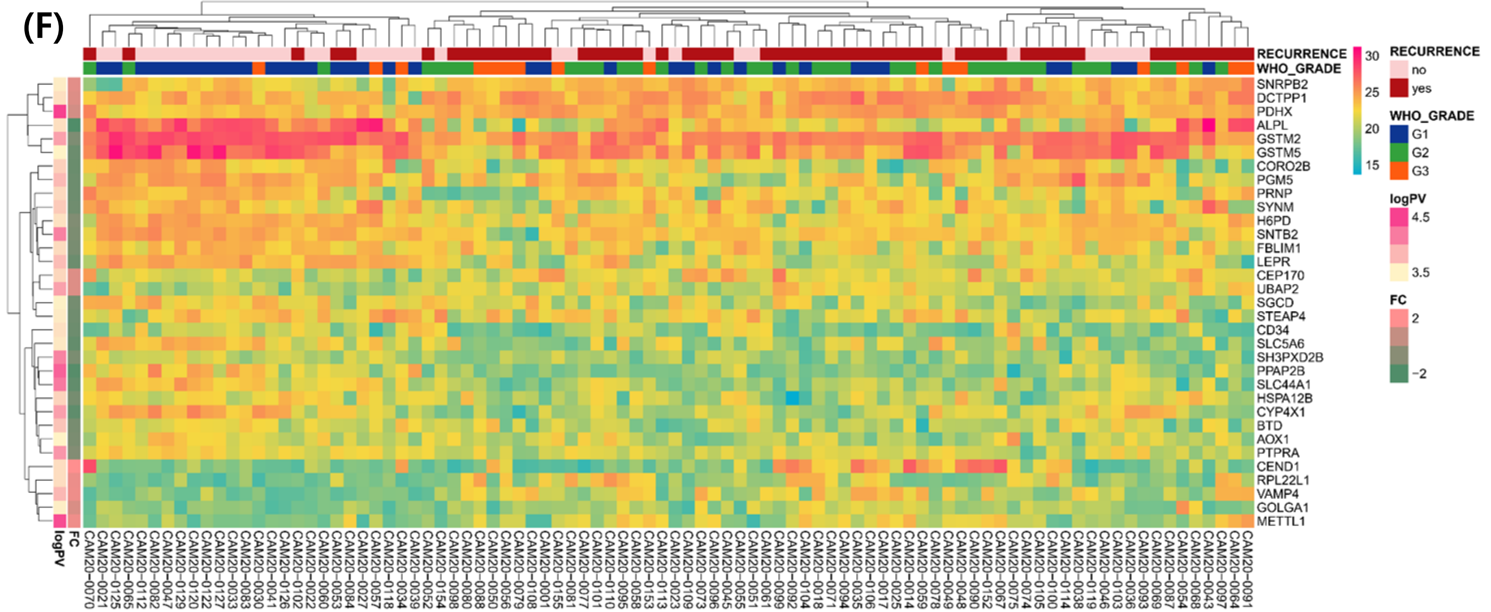

Supplementary Fig. 1

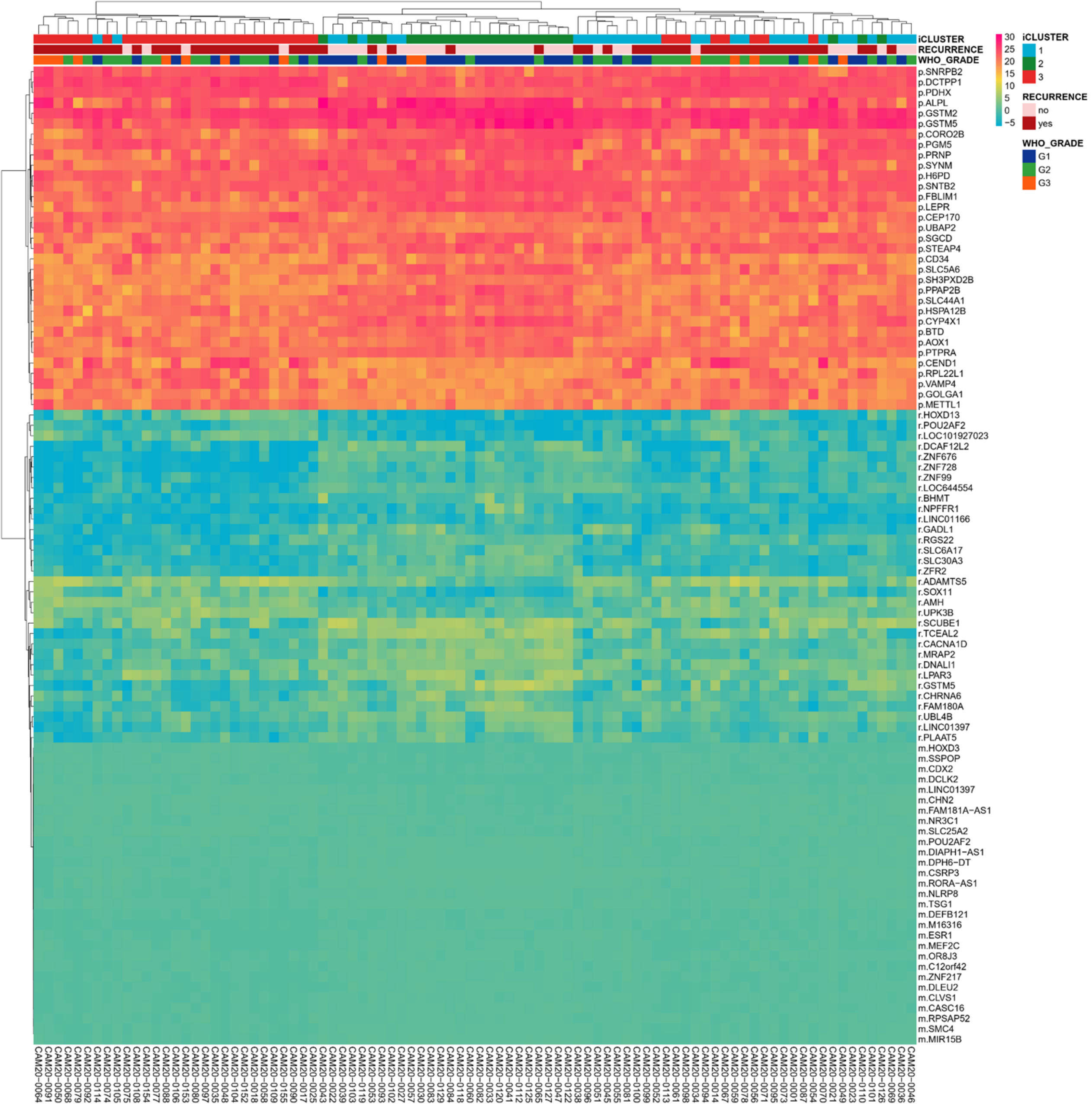

Supplementary Fig. 2

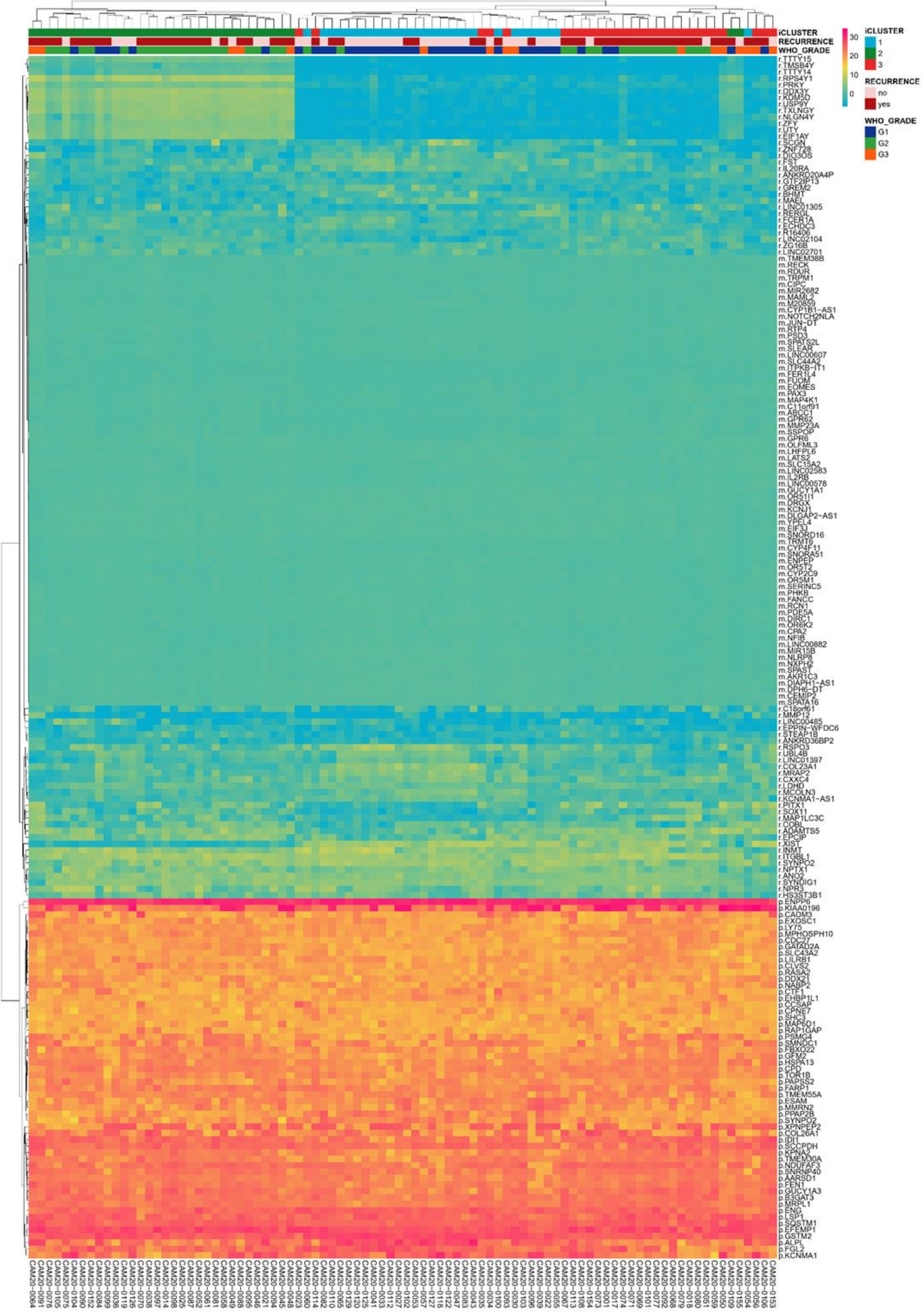

Supplementary Fig. 3

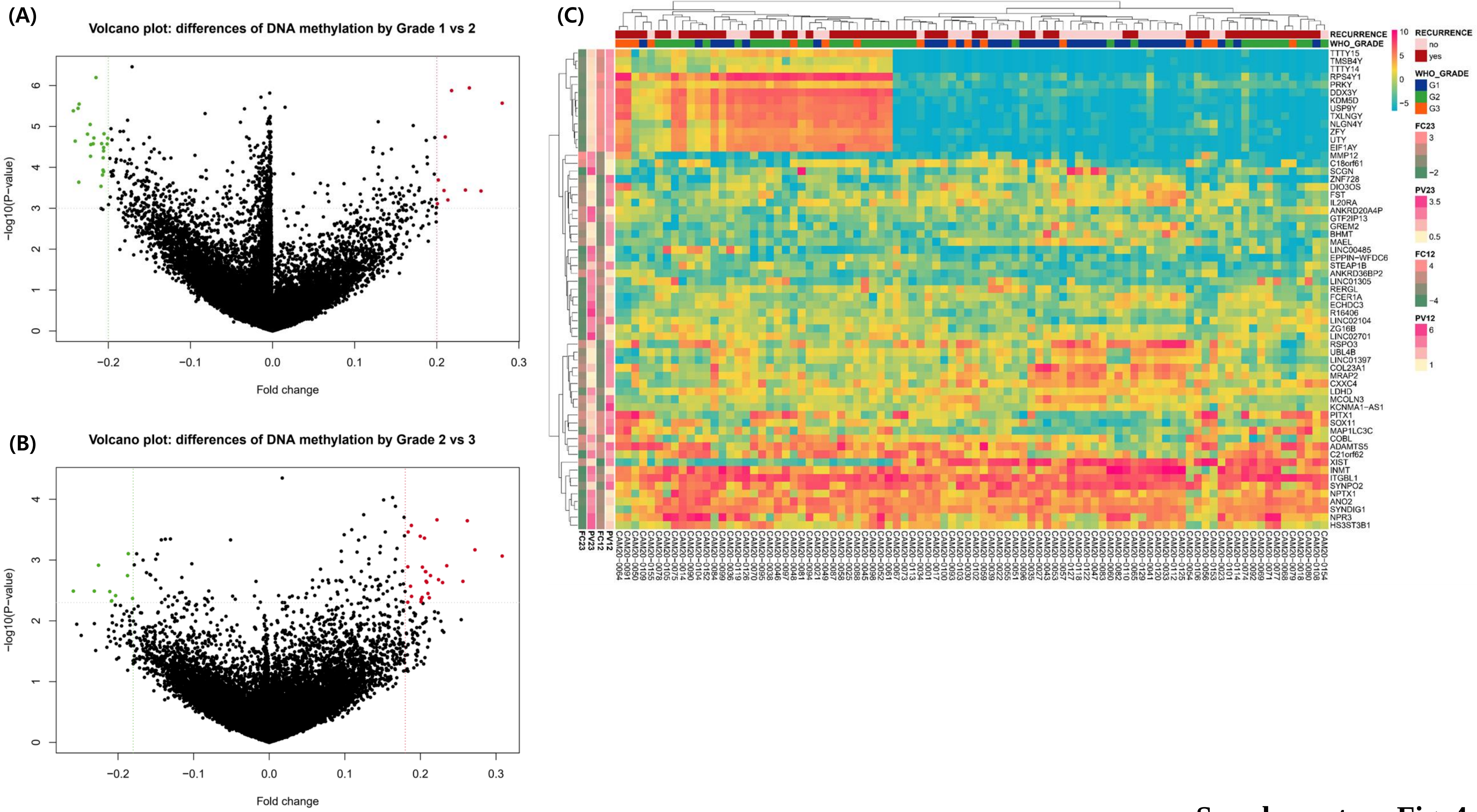

Supplementary Fig. 4

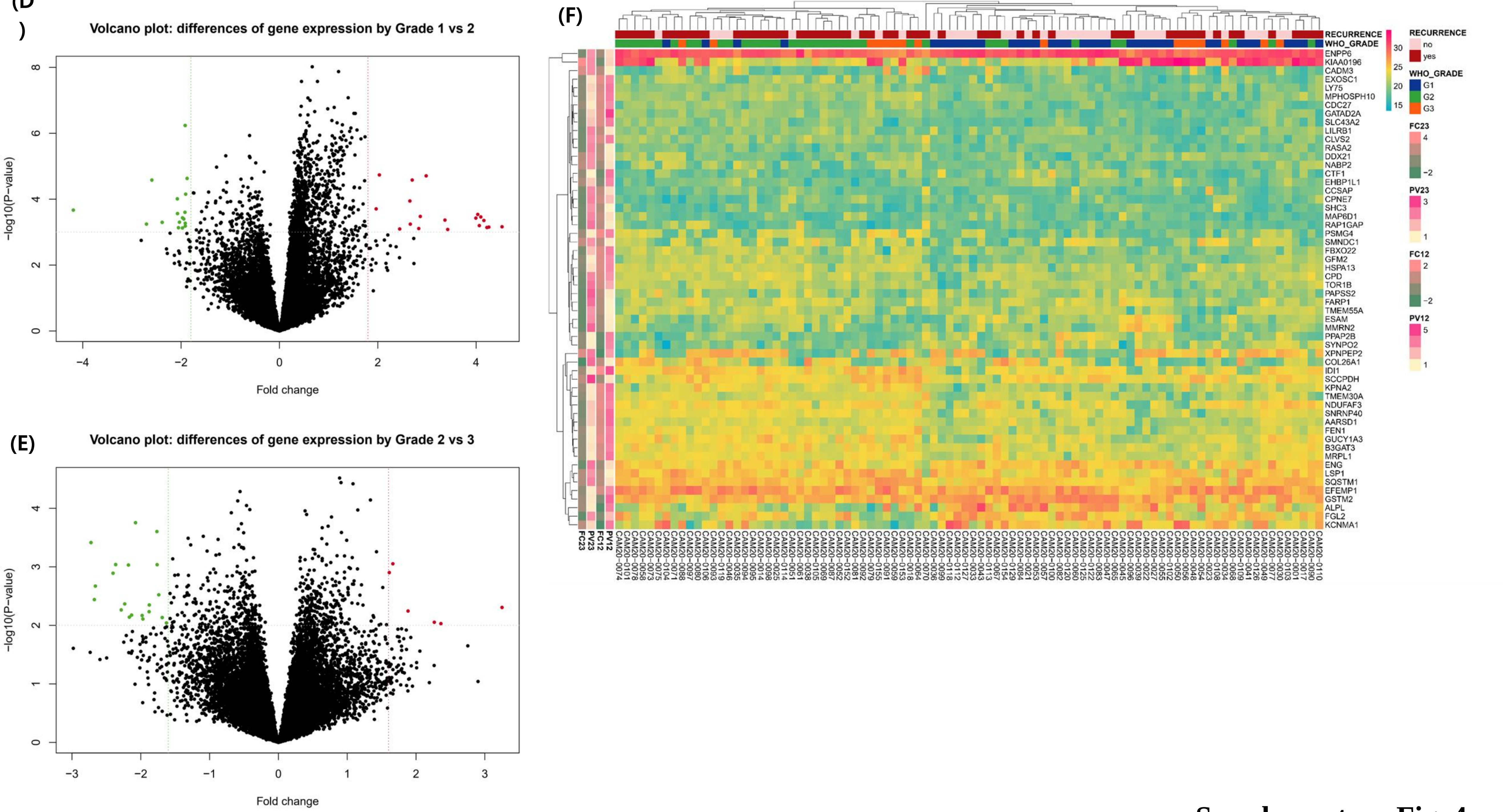

Supplementary Fig. 4

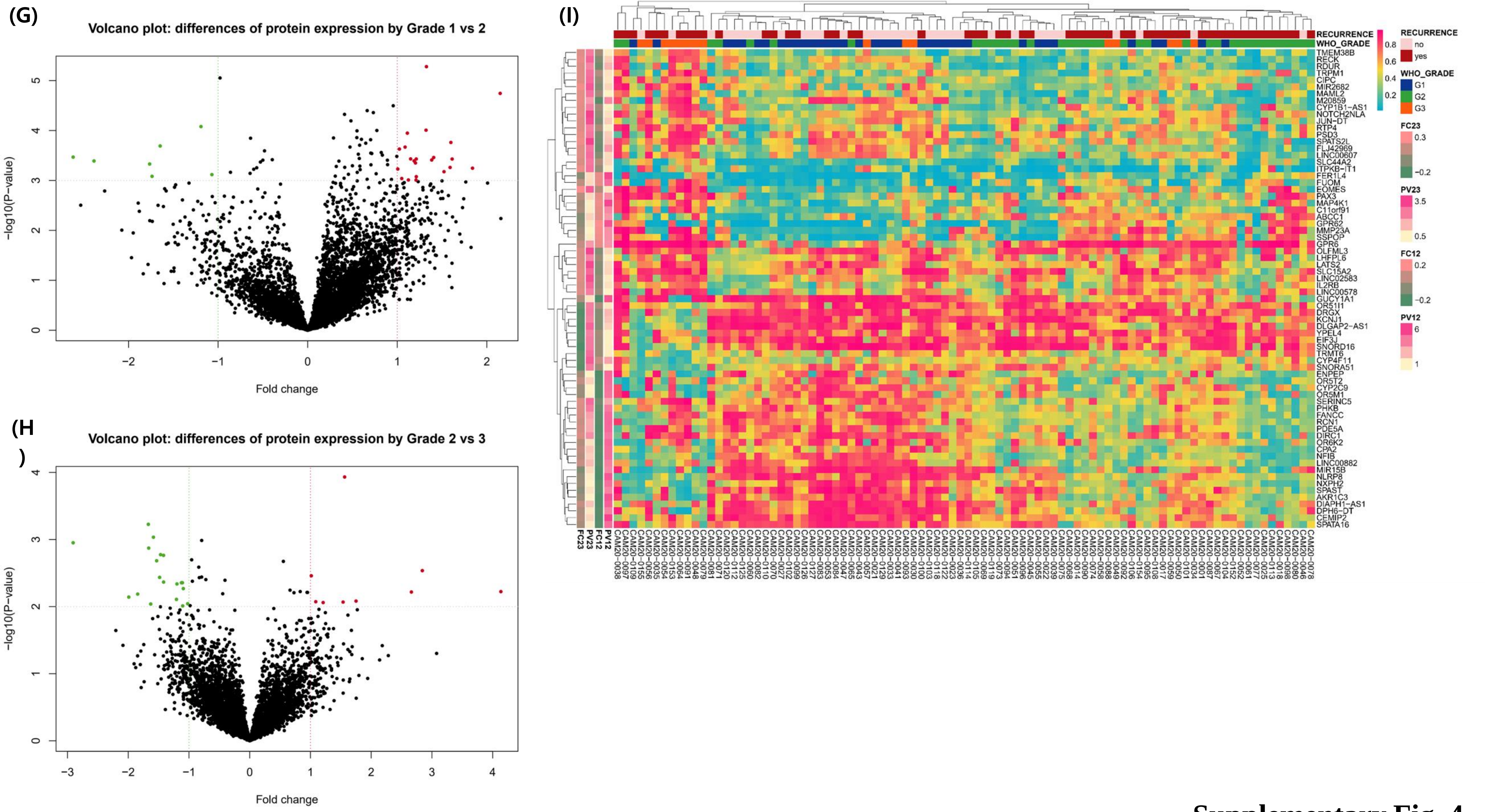

Supplementary Fig. 4

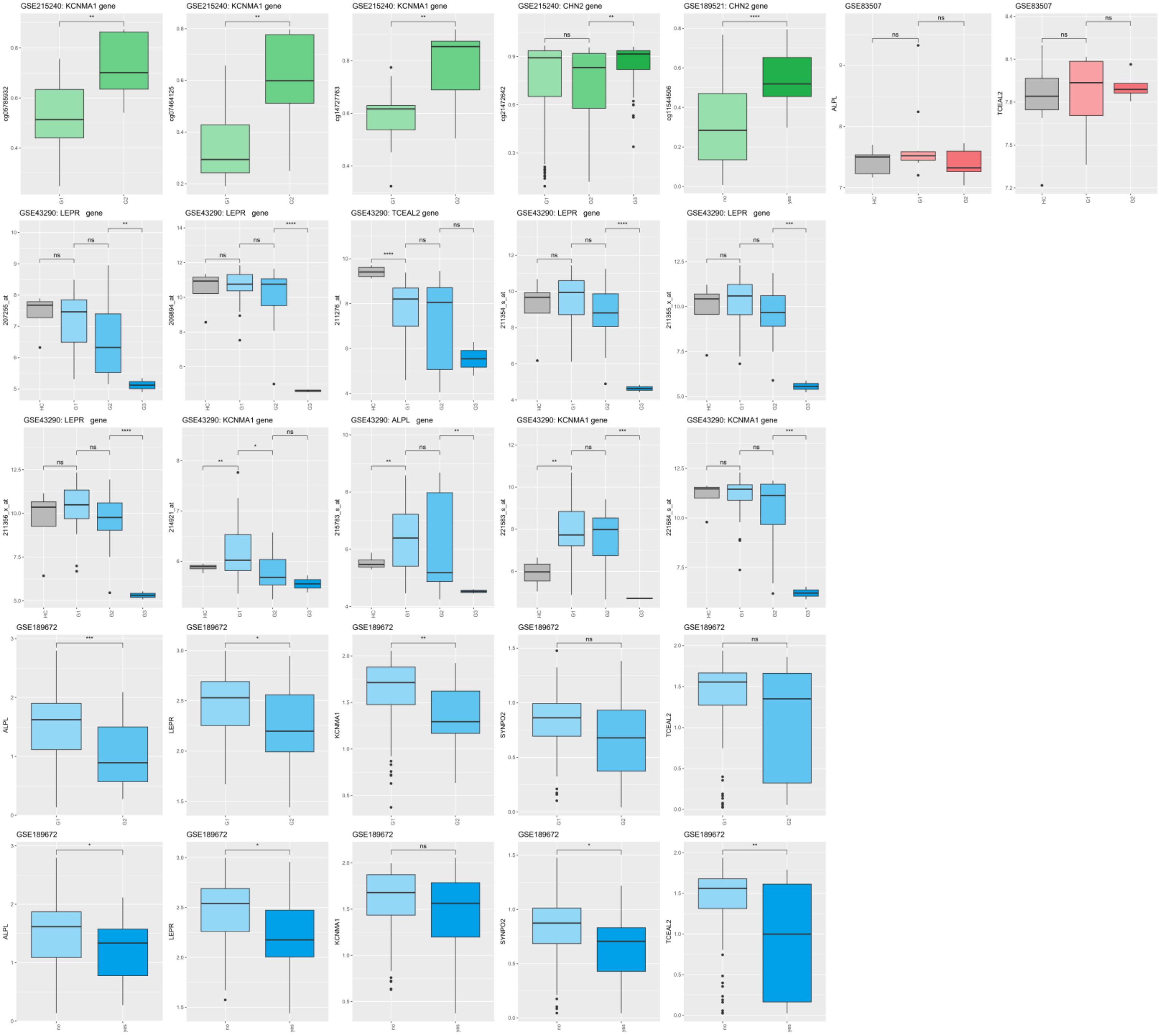

Supplementary Fig. 5

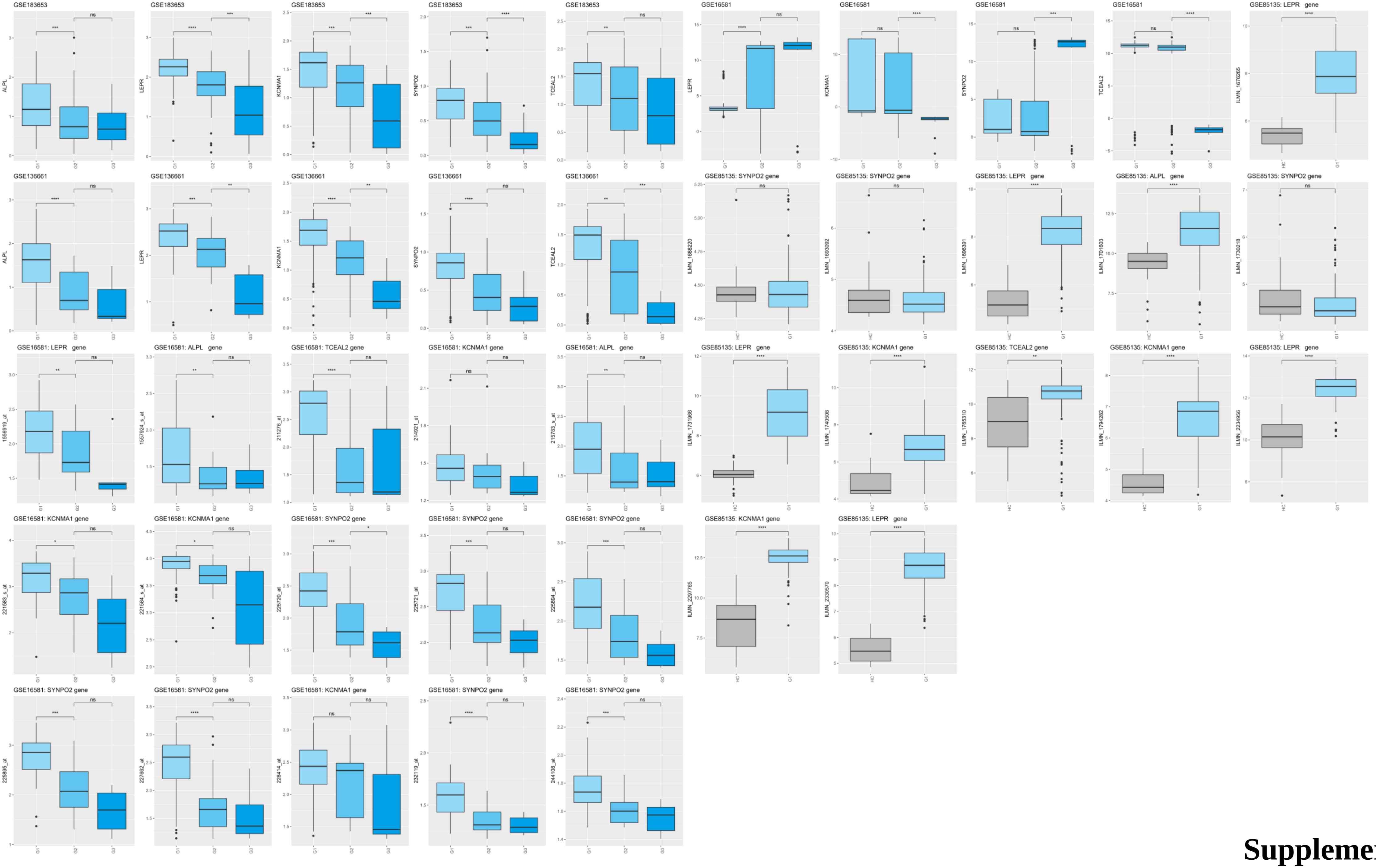

Supplementary Fig. 5
