# Supplementary material for: Network connectome analysis of multi omics data identifies molecular markers of recurrence and grade progression in meningioma
Source: Front Oncol. 2026 Mar 2;16:1745505. doi: 10.3389/fonc.2026.1745505 (PMC12989379; doi:10.3389/fonc.2026.1745505)
Supplement: Supplementary file 2 [file Table1.docx]

**Supplementary Table 1.** Validation of differentially methylated regions (DMRs), differentially expressed genes (DEGs), and differentially expressed proteins (DEPs) on external datasets.

| **Type** | **Acc no.** | **Sample no.** | **Condition** | **Gene no.** | **Overlapped gene list** | | | | | |
| --- | --- | --- | --- | --- | --- | --- | --- | --- | --- | --- |
|  |  |  |  |  | **By recurrence** | | | **By grade** | | |
|  |  |  |  |  | **DMRs** | **DEGs** | **DEPs** | **DMRs** | **DEGs** | **DEPs** |
| Methylation  (Array) | GSE215240 | G1 vs G2  (9 vs 9) | PV < 0.01  \|FC\| > 0.20 | 123 |  |  |  |  |  | KCNMA1,RAP1GAP |
|  | GSE183647 | G1 vs G2  (388 vs 142) | PV < 0.01  \|FC\| > 0.075 | 118 | ZNF217 |  |  |  | SYNDIG1 |  |
|  |  | G2 vs G3  (142 vs 35) | PV < 0.01  \|FC\| > 0.09 | 120 | CHN2 |  |  | EOMES |  |  |
|  | GSE83933 | G1 vs G2  (20 vs 15) | PV < 0.01  \|FC\| > 0.16 | 121 | NR3C1 |  |  |  |  |  |
|  |  | G2 vs G3  (15 vs 4) | PV < 0.01  \|FC\| > 0.33 | 109 |  |  |  |  |  |  |
|  | GSE42882 | G1 vs G2  (9 vs 6) | PV < 0.01  \|FC\| > 0.27 | 108 |  |  |  |  | GREM2 |  |
|  |  | G2 vs G3  (6 vs 4) | PV < 0.01  \|FC\| > 0.65 | 114 |  |  | HSPA12B | RCN1 |  |  |
| Gene expression  (RNA-seq) | GSE183653 | G1 vs G2  (86 vs 74) | PV < 0.01  \|FC\| > 0.21 | 113 |  | GSTM5,TCEAL2 | ALPL,GSTM5,LEPR |  | XIST,RSPO3 | ALPL,ENPP6,KCNMA1 |
|  |  | G2 vs G3  (74 vs 25) | PV < 0.01  \|FC\| > 0.30 | 120 |  |  | AOX1,CYP4X1,LEPR |  | INMT,SYNPO2 | FGL2,KCNMA1,SYNPO2 |
|  | GSE136661 | G1 vs G2  (121 vs 32) | PV < 0.01  \|FC\| > 0.29 | 110 |  | GSTM5,ADAMTS5,UPK3B,MRAP2,TCEAL2 | ALPL,GSTM5,LEPR,RPL22L1 |  | XIST,ADAMTS5,COL23A1,MRAP2,SYNPO2 | ALPL,KCNMA1,SYNPO2 |
|  |  | G2 vs G3  (32 vs 7) | PV < 0.01  \|FC\| > 0.45 | 112 |  | TCEAL2 | LEPR | NXPH2 | RPS4Y1,DDX3Y,EIF1AY | ENPP6,FGL2,KCNMA1 |
| Gene expression  (Array) | GSE16581 | G1 vs G2  (86 vs 74) | PV < 0.01  \|FC\| > 0.35 | 125 | CHN2 | GSTM5,MRAP2,TCEAL2 | ALPL,GSTM5,STEAP4 |  | INMT,RSPO3,MRAP2,SYNPO2 | ALPL,SYNPO2 |
|  |  | G2 vs G3  (74 vs 25) | PV < 0.01  \|FC\| > 0.29 | 124 |  |  |  | RECK | KDM5D,USP9Y,DDX3Y,EIF1AY,NLGN4Y,C21orf62,TXLNGY |  |
|  | GSE16156 | G1 vs G2  (105 vs 66) | PV < 0.01  \|FC\| > 2.5 | 125 |  | SOX11 | LEPR |  | SOX11,MCOLN3 |  |
|  |  | G2 vs G3  (66 vs 18) | PV < 0.01  \|FC\| > 1.5 | 110 |  | SOX11,TCEAL2 |  |  | SOX11,INMT,MCOLN3,SYNPO2 | KCNMA1,SYNPO2 |
|  |  | Rx vs Ro  (124 vs 65) | PV < 0.01  \|FC\| > 2.5 | 114 |  | SOX11 | LEPR |  | SOX11,MCOLN3 |  |
|  | GSE85135 | HC vs G1  (25 vs 96) | PV < 0.01  \|FC\| > 2.8 | 123 |  |  | LEPR |  |  | ENPP6,KCNMA1 |
|  | GSE43290 | G1 vs G23  (33 vs 14) | PV < 0.01  \|FC\| > 0.38 | 108 | SMC4 |  | AOX1 | RCN1 | ITGBL1 | FGL2,KCNMA1,KPNA2 |
|  |  | HC vs G1  (4 vs 33) | PV < 0.01  \|FC\| > 3 | 112 |  |  |  |  | RERGL |  |
|  |  | HC vs G23  (4 vs 14) | PV < 0.01  \|FC\| > 3 | 111 |  |  |  |  | RERGL |  |
| Protein expression | GSE83507 | HC vs G1  (15 vs 10) | PV < 0.01  \|FC\| > 0.62 | 120 |  |  |  |  | FST |  |
|  |  | HC vs G2  (15 vs 5) | PV < 0.01  \|FC\| > 0.52 | 125 |  |  |  |  | FST |  |
|  |  | G1 vs G2  (10 vs 5) | PV < 0.01  \|FC\| > 0.15 | 124 |  |  |  |  |  |  |
| Methylation and expression | GSE189521 | G1 vs G2  (90 vs 20) | PV < 0.01  \|FC\| > 0.210 | 105 |  | AMH |  |  |  |  |
|  |  | Rx vs Ro  (91 vs 19) | PV < 0.01  \|FC\| > 0.215 | 100 | CHN2,CASC16 |  |  | FANCC,ABCC1 |  |  |
|  | GSE189672 | G1 vs G2  (90 vs 20) | PV < 0.01  \|FC\| > 0.21 | 121 |  | UPK3B,MRAP2 | ALPL,RPL22L1 |  | XIST,CXXC4,COL23A1,MRAP2 | ALPL,KCNMA1,PAPSS2 |
|  |  | Rx vs Ro  (91 vs 19) | PV < 0.01  \|FC\| > 0.20 | 122 |  | LPAR3,TCEAL2,GADL1,FAM180A |  |  | FCER1A,RSPO3 |  |
